# Supplementary material for: Ring-Finger Protein 126 (RNF126) Promotes Anoikis Resistance and Peritoneal Colonization in Ovarian Cancer
Source: Int J Mol Sci. 2025 Dec 18;26(24):12183. doi: 10.3390/ijms262412183 (PMC12734305; doi:10.3390/ijms262412183)
Supplement: Supplementary file 1 [file ijms-26-12183-s001.zip › Figure_S2.pdf]

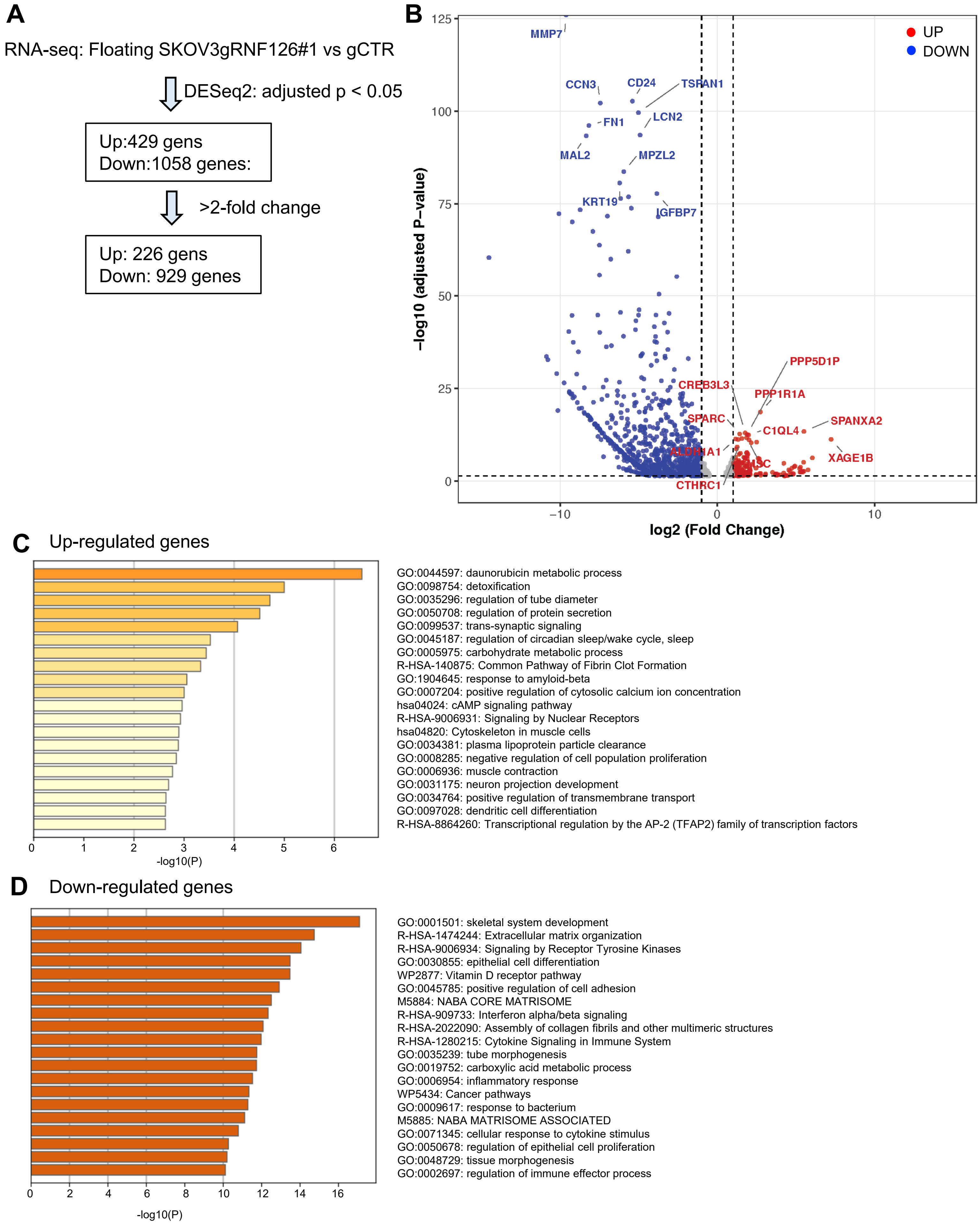
A
RNA-seq: Floating SKOV3gRNF126#1 vs gCTR
DESeq2: adjusted p < 0.05
Up:429 gens
Down:1058 genes:
>2-fold change
Up: 226 gens
Down: 929 genes
B
UP
DOWN
MMP7
CCN3
CD24
TSPAN1
FN1
LCN2
MAL2
MPZL2
KRT19
IGFBP7
PPP5D1P
CREB3L3
PPP1R1A
SPARC
C1QL4
SPANXA2
ALDH1A1
XAGE1B
MSC
CTHRC1
−log10 (adjusted P−value)
log2 (Fold Change)
125
100
75
50
25
0
−10
0
10
C
Up-regulated genes
GO:0044597: daunorubicin metabolic process
GO:0098754: detoxification
GO:0035296: regulation of tube diameter
GO:0050708: regulation of protein secretion
GO:0099537: trans-synaptic signaling
GO:0045187: regulation of circadian sleep/wake cycle, sleep
GO:0005975: carbohydrate metabolic process
R-HSA-140875: Common Pathway of Fibrin Clot Formation
GO:1904645: response to amyloid-beta
GO:0007204: positive regulation of cytosolic calcium ion concentration
hsa04024: cAMP signaling pathway
R-HSA-9006931: Signaling by Nuclear Receptors
hsa04820: Cytoskeleton in muscle cells
GO:0034381: plasma lipoprotein particle clearance
GO:0008285: negative regulation of cell population proliferation
GO:0006936: muscle contraction
GO:0031175: neuron projection development
GO:0034764: positive regulation of transmembrane transport
GO:0097028: dendritic cell differentiation
R-HSA-8864260: Transcriptional regulation by the AP-2 (TFAP2) family of transcription factors
0
1
2
3
4
5
6
-log10(P)
D
Down-regulated genes
GO:0001501: skeletal system development
R-HSA-1474244: Extracellular matrix organization
R-HSA-9006934: Signaling by Receptor Tyrosine Kinases
GO:0030855: epithelial cell differentiation
WP2877: Vitamin D receptor pathway
GO:0045785: positive regulation of cell adhesion
M5884: NABA CORE MATRISOME
R-HSA-909733: Interferon alpha/beta signaling
R-HSA-2022090: Assembly of collagen fibrils and other multimeric structures
R-HSA-1280215: Cytokine Signaling in Immune System
GO:0035239: tube morphogenesis
GO:0019752: carboxylic acid metabolic process
GO:0006954: inflammatory response
WP5434: Cancer pathways
GO:0009617: response to bacterium
M5885: NABA MATRISOME ASSOCIATED
GO:0071345: cellular response to cytokine stimulus
GO:0050678: regulation of epithelial cell proliferation
GO:0048729: tissue morphogenesis
GO:0002697: regulation of immune effector process
0
2
4
6
8
10
12
14
16
-log10(P)

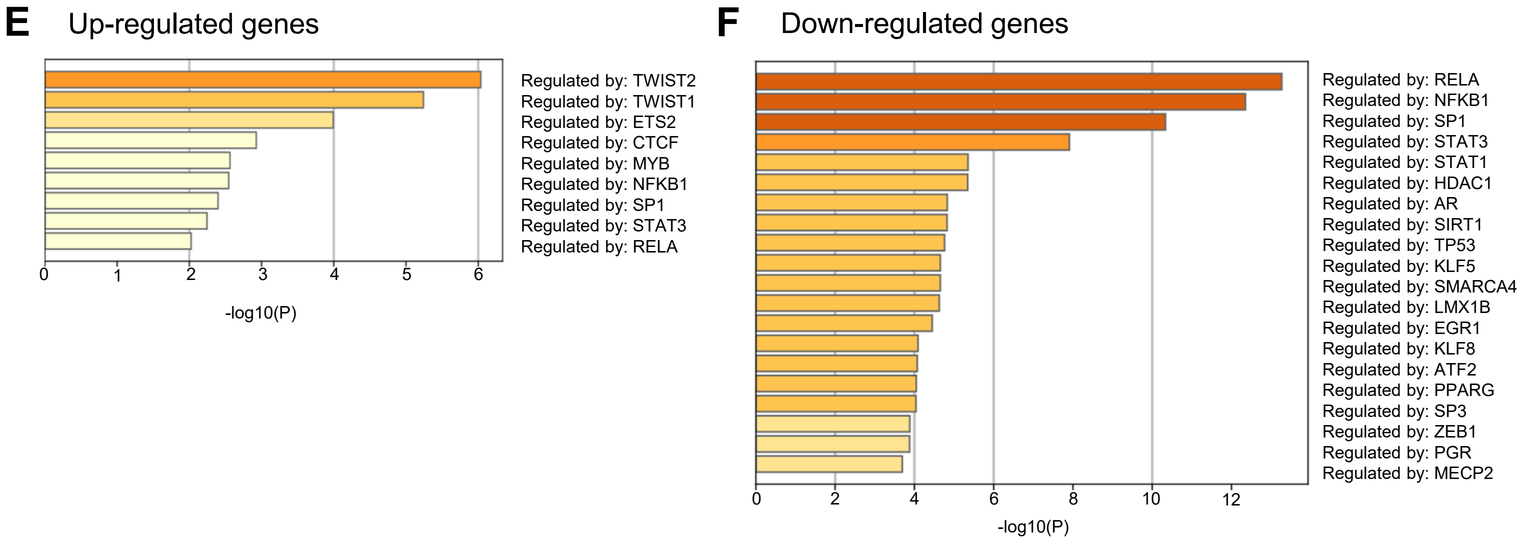

Figure S2. RNF126 depletion alters the transcriptome of SKOV3 cells cultured in floating conditions. (**A**) Schemes of transcriptome data analyses of control and RNF126-depleted SKOV3 cells under floating conditions. **(B)** Volcano plot of DEGs between control and RNF126-depleted SKOV3 cells under floating conditions. (**C, D**) Enriched gene ontology of up-regulated (**C**) and down-regulated (**D**) genes by RNF126 depletion. (**E, F**) Enriched transcriptional factors that target up-regulated (**E**) and down-regulated (**F**) genes by RNF126 depletion.
