# Supplementary figures and images for: Ring-Finger Protein 126 (RNF126) Promotes Anoikis Resistance and Peritoneal Colonization in Ovarian Cancer
Source: Int J Mol Sci. 2025 Dec 18;26(24):12183. doi: 10.3390/ijms262412183 (PMC12734305; doi:10.3390/ijms262412183)

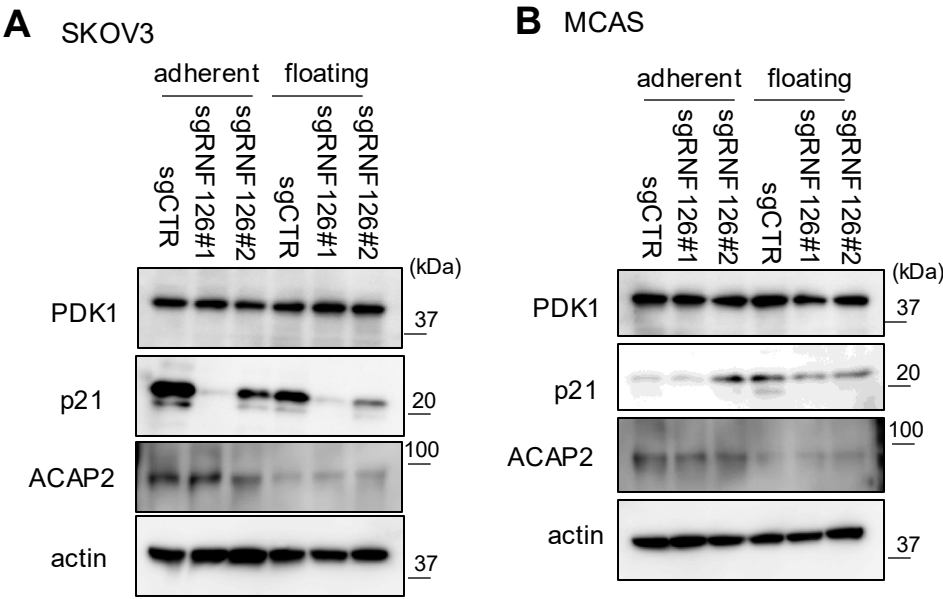

**Figure S1.** Western blotting of p21, PDK1, and ACAP2 in control and RNF126-depleted SKOV3(A) and MCAS(B) cells

Supplement: Supplementary file 1 [file ijms-26-12183-s001.zip › Figure_S1.pdf]
